# Comparison of procedural efficacy and biophysical parameters between two competing cryoballoon technologies for pulmonary vein isolation: Insights from an initial multicenter experience

**DOI:** 10.1111/jce.14915

**Published:** 2021-02-01

**Authors:** Sing‐Chien Yap, Ante Anic, Toni Breskovic, Annika Haas, Rohit E. Bhagwandien, Zrinka Jurisic, Tamas Szili‐Torok, Armin Luik

**Affiliations:** ^1^ Department of Cardiology, Erasmus MC University Medical Center Rotterdam Rotterdam The Netherlands; ^2^ Department of Cardiology Clinical Hospital Center Split Split Croatia; ^3^ Medizinische Klinik IV, Städtisches Klinikum Karlsruhe Academic Teaching Hospital of the University of Freiburg Karlsruhe Germany

**Keywords:** atrial fibrillation, catheter ablation, cryoablation, cryoballoon, pulmonary vein isolation

## Abstract

**Introduction:**

Recently a novel cryoballoon system (POLARx, Boston Scientific) became available for the treatment of atrial fibrillation. This cryoballoon is comparable with Arctic Front Advance Pro (AFA‐Pro, Medtronic), however, it maintains a constant balloon pressure. We compared the procedural efficacy and biophysical characteristics of both systems.

**Methods:**

One hundred and ten consecutive patients who underwent first‐time cryoballoon ablation (POLARx: *n* = 57; AFA‐Pro: *n* = 53) were included in this prospective cohort study.

**Results:**

Acute isolation was achieved in 99.8% of all pulmonary veins (POLARx: 99.5% vs. AFA‐Pro: 100%, *p* = 1.00). Total procedure time (81 vs. 67 min, *p* < .001) and balloon in body time (51 vs. 35 min, *p* < .001) were longer with POLARx. After a learning curve, these times were similar. Cryoablation with POLARx was associated with shorter time to balloon temperature −30°C (27 vs. 31 s, *p* < .001) and −40°C (32 vs. 54 s, *p* < .001), lower balloon nadir temperature (−55°C vs. −47°C, *p* < .001), and longer thawing time till 0°C (16 vs. 9 s, *p* < .001). There were no differences in time‐to‐isolation (TTI; POLARx: 45 s vs. AFA‐Pro 43 s, *p* = .441), however, POLARx was associated with a lower balloon temperature at TTI (−46°C vs. −37°C, *p* < .001). Factors associated with acute isolation differed between groups. The incidence of phrenic nerve palsy was comparable (POLARx: 3.5% vs. AFA‐Pro: 3.7%).

**Conclusion:**

The novel cryoballoon is comparable to AFA‐Pro and requires only a short learning curve to get used to the slightly different handling. It was associated with faster cooling rates and lower balloon temperatures but TTI was similar to AFA‐Pro.

**Abbreviations**
AF: atrial fibrillation
AFA‐Pro: Arctic Front Advance Pro
CB: cryoballoon
CBA: cryoballoon application
DMS: diaphragmatic movement sensor
DOAC: direct oral anticoagulant
PV: pulmonary vein
PVI: pulmonary vein isolation
TTI: time to isolation

## 1 INTRODUCTION

The cornerstone of atrial fibrillation (AF) ablation is complete isolation of the pulmonary veins (PVs).^1^ Among the different available single‐shot devices, the cryoballoon has demonstrated to be as effective and safe as radiofrequency ablation for achieving pulmonary vein isolation (PVI), while being associated with shorter procedure duration and longer fluoroscopy time.^2^, ^3^, ^4^, ^5^, ^6^, ^7^ Furthermore, cryoballoon ablation seems to be less operator‐dependent than radiofrequency ablation.^8^ Recently, a novel cryoballoon was introduced, the POLARx cryoablation system (Boston Scientific). The unique feature of this cryoballoon is that it maintains a uniform pressure and size during inflation and cryoablation. Theoretically, a more compliant balloon can improve PV occlusion resulting in a more effective cryoenergy delivery. Currently, limited data exists on the biophysical characteristics of this novel cryoballoon.^9^ Knowledge of biophysical parameters, such as nadir balloon temperature and balloon thawing time, is important as they have been shown to be associated with durability of PVI after cryoballoon ablation.^10^, ^11^, ^12^

### 1.1 Aim of the study

The aim of this study was to compare the procedural efficacy and biophysical parameters of the novel POLARx system (Boston Scientific) with the currently established fourth‐generation Arctic Front Advance Pro system (AFA‐Pro, Medtronic).

## 2 METHODS

### 2.1 Study population

In this prospective cohort study, we included consecutive patients who underwent a first‐time cryoballoon ablation for the treatment of symptomatic paroxysmal or persistent AF between May and October 2020. Starting in May 2020, there was a limited market release of the POLARx cryoablation system in Europe. Patients were included from three highly experienced cryoballoon ablation centers. The study was approved by the institutional review board of each center.

### 2.2 Periprocedural management

All patients received oral anticoagulation for at least 4 weeks before ablation. Periprocedural anticoagulation regime was carried out according to the local standards. To exclude left atrial thrombi, all patients underwent transesophageal echocardiogram within 24 h of the procedure or by intracardiac echocardiography before transseptal puncture.

### 2.3 Ablation procedure

**Figure 1:**
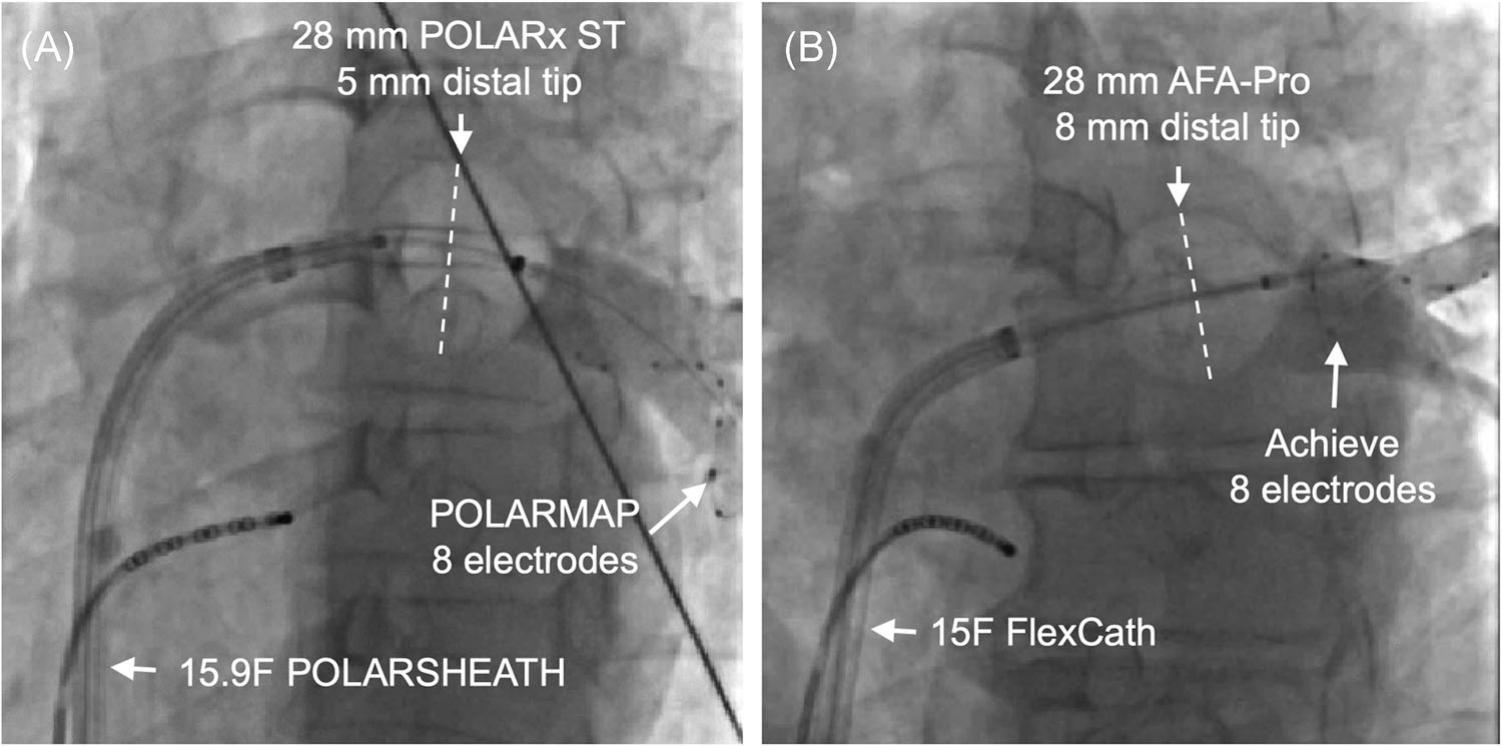
Cine‐images of both cryoballoons during PV occlusion. (A) 28‐mm POLARx cryoballoon with 5 mm tip, POLARMAP circular mapping catheter and 15.9F POLARSHEATH; (B) 28‐mm AFA‐Pro cryoballoon with 8 mm tip, Achieve circular mapping catheter and 15F FlexCath

All procedures were performed with local anesthesia and analgosedation. After vascular access was obtained, a single transseptal puncture was performed. Intravenous heparin was administered to achieve a target activated clotting time of more than or equal to 300 s. All patients underwent PVI using a 28‐mm cryoballoon (AFA‐Pro [8‐mm tip], Medtronic; or POLARx [short tip: 5‐mm tip or long tip: 12‐mm tip, Boston Scientific]; Figure 1). The balloon was inserted through a steerable sheath (15‐F FlexCath, Medtronic; or 15.9‐F POLARSHEATH, Boston Scientific). PV potentials were recorded using a 20‐mm circular inner lumen mapping catheter with 8 electrodes (Achieve, Medtronic; or POLARMAP, Boston Scientific). After optimal PV occlusion was achieved, assessed by contrast injection, cryoablation was started. A time‐to‐isolation (TTI) guided ablation protocol was used. The freeze duration was 180 s if TTI was less than 60 s, otherwise a 240‐s freeze cycle was employed. No bonus freeze was employed routinely. PVI was confirmed by entrance/exit block at the end of the procedure.

During cryoablation of the right‐sided PVs, either breathing maneuvers or high‐output right phrenic nerve stimulation was performed using a diagnostic catheter in the right subclavian vein or superior vena cava. Diaphragmatic excursion was assessed by palpation or, in case of the POLARx system, by using the Diaphragmatic Movement Sensor (DMS). The DMS uses an accelerometer and provides a relative measure of the diaphragmatic excursion. Whenever the diaphragmatic excursions decreased or the DMS percentage drops below a cutoff (65%), cryoablation was immediately terminated. During cryoablation of the left‐sided PVs, a diagnostic catheter was placed in the right ventricle to provide ventricular pacing in case of a vagal response after cryoablation.

### 2.4 Data collection

Patient demographic and clinical data were obtained from the medical records. A case report form was used to capture all relevant procedural and biophysical data. For every cryoballoon application (CBA) the following parameters were collected: grade of PV occlusion (Grades 1–4),^13^ the presence of PV potential during ablation, duration of CBA, TTI (if measurable), balloon temperature at TTI, time to balloon temperature −30°C, time to balloon temperature −40°C, balloon nadir temperature, and thawing time till 0°C. The last parameter was defined as the time needed for the balloon to rewarm till 0°C upon completion of the CBA. This thawing parameter was chosen because a previous study demonstrated that a thawing time till 0°C more than or equal to 10 s predicts durable PVI after cryoballoon ablation with the second‐generation cryoballoon (Arctic Front Advance, Medtronic).^10^ TTI was defined as the time duration required to achieve PVI after start of CBA. All time variables were expressed in seconds.

With the AFA‐Pro cryoballoon system, TTI was manually recorded after achieving PVI. The cryoablation binary data files stored in the CryoConsole (Medtronic) were used to analyze various biophysical parameters (i.e., balloon temperature at TTI, time to balloon temperature −30°C, time to balloon temperature −40°C, nadir balloon temperature, and thawing time till 0°C).

With the POLARx cryoballoon system, TTI could be annotated by the operator during the CBA by pressing the foot pedal for 3 s. Most biophysical data can be exported from the SMARTFREEZE console (Boston Scientific) onto a pdf‐file (i.e., TTI, time to balloon temperature −30°C, time to balloon temperature −40°C, nadir balloon temperature, and thawing time till 0°C). Only balloon temperature at TTI had to be collected from the cryoablation binary data files from the SMARTFREEZE console (Boston Scientific).

### 2.5 Follow‐up

All patients were followed up for at least 1 month after the procedure to collect data on acute procedural complications and mainly to rule out atrioesophageal fistula. Patients with phrenic nerve palsy underwent a chest x‐ray during follow‐up. Patients who developed pulmonary symptoms underwent a cardiac computed tomography to rule out complications. Antiarrhythmic drugs were stopped at the discretion of the operator.

### 2.6 Statistical method

Continuous data are presented as median with 25th and 75th percentile as the data were not normally distributed. Categorical variables are presented by frequencies and percentages. Differences of continuous variables between the two groups were analyzed with the nonparametric Mann–Whitney *U*‐test. Differences between categorical variables were evaluated using the *χ* ^2^ test or Fisher′s exact test. Statistical analyses were performed using MATLAB R2020a. All statistical tests were two‐sided. *p* values less than .05 were considered statistically significant.

## 3 RESULTS

### 3.1 Study population

In the study period, 110 consecutive patients underwent a first cryoballoon ablation for the treatment of AF. The POLARx and AFA‐Pro system was used in 57 and 53 patients, respectively. The short‐tip POLARX balloon was used in the majority (93.0%) of the cases in the POLARx group. Baseline patient characteristics are presented in Table 1. The POLARx group had a lower proportion of patients with hypertension and a higher proportion of patients using a DOAC in comparison with the AFA‐Pro group. The use of antiarrhythmic drug therapy was more common in the AFA‐Pro group. Other baseline variables were similar between groups, including age, sex, type of AF, CHA_2_DS_2_‐VASc score, left ventricular ejection fraction, and left atrial dimension. The majority of patients had paroxysmal AF (75.5%).

**Table 1:** Baseline patient characteristics

Variables	POLARx (*n* = 57)	AFA‐Pro (*n* = 53)	*p*‐value
Age, years	61 (57, 66)	64 (57, 70)	.082
Male sex	33 (57.9%)	36 (67.9%)	.326
Hypertension	18 (31.6%)	31(58.5%)	.007
Diabetes	3 (5.3%)	3 (5.6%)	1.000
Coronary artery disease	8 (14.0%)	5 (9.4%)	.560
CABG	2 (3.5%)	1 (1.9%)	1.000
Paroxysmal AF	43 (75.4%)	40 (75.5%)	1.000
LVEF (%)	63 (60, 65)	60 (60, 65)	.813
LA diameter, mm	41 (36, 44)	41 (37, 43)	.732
CHA_2_DS_2_‐VASc score	1 (1, 2)	2 (0, 3)	.533
Vitamin K antagonist		3 (5.7%)	.109
DOAC	57 (100.0%)	48 (90.6%)	.023
Antiarrhythmic drugs	30 (52.6%)	40 (75.5%)	.017

*Note*: Values are presented as median (25th, 75th percentile) or as count (%).

Abbreviations: AF, atrial fibrillation; AFA‐Pro, Arctic Front Advance Pro; CABG, coronary artery bypass graft; DOAC, direct‐acting oral anticoagulant; LA, left atrium; LVEF, left ventricular ejection fraction.

### 3.2 Procedural efficacy

A total of 422 PVs was targeted (POLARx: *n* = 216, AFA‐Pro: *n* = 206). Acute isolation was achieved in 99.8% of all PVs, and was similar between groups (POLARx: 99.5% vs. AFA‐Pro: 100%, *p* = 1.00). One left superior PV (diameter of 15 mm on CT‐scan) could not be isolated in the POLARx group despite four CBAs with Grade 4 occlusion. Procedure‐related variables are presented in Table 2. Procedure time and balloon in body time were longer, and the amount of contrast agent used was higher in the POLARx group in comparison with the AFA‐Pro group. Other procedure‐related variables, including the median number of CBA per patient, fluoroscopy time, radiation dose, and additional CTI ablation were similar between groups (Table 2).

**Table 2:** Procedural and clinical outcomes

Variables	POLARx (*n* = 57)	AFA‐Pro (*n* = 53)	*p*‐value
Procedural			
Procedure time, min	81 (70, 95)	67 (49, 83)	<.001
Balloon in body time, min	51 (41, 62)	35 (31, 42)	<.001
Fluoroscopy time, min	14.0 (9.8, 18.3)	10.8 (8.1, 16.1)	.141
Radiation dose, cGy*cm^2^	637 (375, 1133)	686 (358, 1083)	.817
Contrast agent, ml	50 (40, 60)	40 (25, 50)	.002
Number of CBA per patient	5 (4, 6)	5 (4, 6)	.339
Left common ostium PV	12 (23.1%)	6 (12.5%)	.203
Total duration CBA, s	995 (870, 1.262)	912 (821, 1.173)	.277
Duration CBA LSPV, s	209 (180, 289)	180(180, 240)	.553
Duration CBA LIPV, s	180 (180, 240)	180 (180, 249)	.500
Duration CBA RSPV, s	240 (180, 277)	180 (180, 240)	.466
Duration CBA RIPV, s	180 (180, 240)	180 (180, 240)	.171
Duration CBA LCPV, s	240 (180, 240)	240 (210, 367)	.373
Additional CTI ablation	3 (5.3%)	7 (13.2%)	.192
Acute procedural complications			
Groin hematoma	1 (1.8%)	0 (0%)	1.000
Phrenic nerve palsy	2 (3.5%)	2 (3.7%)	1.000
TIA	1 (1.8%)	0 (0%)	1.000

*Note* Values are presented as median (25th, 75th percentile) or as count (%).

Abbreviations: AF, atrial fibrillation; AFA‐Pro, Arctic Front Advance Pro; CBA, cryoballoon application; CTI, cavotricuspid isthmus; LIPV, left inferior pulmonary vein; LSPV, left superior pulmonary vein; PV, pulmonary vein, RIPV, right inferior pulmonary vein; RSPV, right superior pulmonary vein; TIA, transient ischemic attack.

A learning curve analysis was performed with regard to procedural parameters. Analysis of the second half of the cohort showed no difference in procedure time (POLARx: 78 [63–95] min vs. AFA‐Pro: 75 [53–85] min, *p* = .149), balloon in body time (POLARx: 43 [38–61] min vs. AFA‐Pro: 38 [31–44] min, *p* = .066), and contrast dye usage (POLARx: 50 [40–65] ml vs. AFA‐Pro: 43 [36–50] ml, *p* = .063).

### 3.3 Comparison of procedural and biophysical parameters between groups

**Figure 2:**
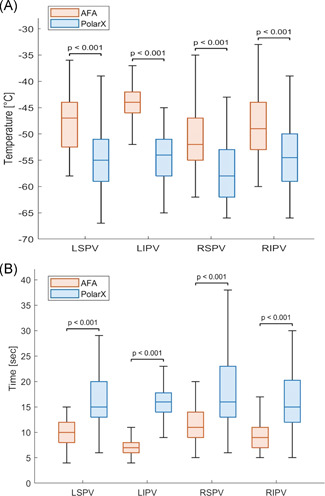
Balloon nadir temperature (A) and thawing time till 0°C (B) per pulmonary vein. AFA, Arctic Front Advance; LIPV, left inferior pulmonary vein; LSPV, left superior pulmonary vein; RIPV, right inferior pulmonary vein; RSPV, right superior pulmonary vein

There was no difference in the magnitude of PV occlusion between groups (Table 3). In the majority of cases a Grade 4 occlusion could be achieved. Cryoablation with POLARx was associated with a shorter time to balloon temperature −30°C and −40°C, a lower balloon nadir temperature, and a longer thawing time till 0°C. PV potentials could be recorded more often during CBA with POLARx than with AFA‐Pro (96.3% vs. 88.6%, *p* < .001). TTI could be recorded in 93.1% of PVs using POLARx versus 79.6% using AFA‐Pro (*p* < .001). There were no differences in TTI between systems, however, POLARx was associated with a lower balloon temperature at TTI in comparison with AFA‐Pro. Detailed information with regard to balloon nadir temperature and thawing time till 0°C for each PV is presented in Figure 2. POLARx was associated with a lower balloon nadir temperature and longer thawing time till 0°C for each PV in comparison with AFA‐Pro.

**Table 3:** Comparison of procedural and biophysical parameters between POLARx and AFA‐Pro

Variables	POLARx	AFA‐Pro	*p*‐value
Total number of CBA^a^	299	264	
Grade of PV occlusion			.206
Grade 4	244 (81.6%)	204 (77.3%)	
Grade 3	52 (17.4%)	57 (21,6%)	
Grade 2	3 (1.0%)	3 (1.1%)	
Time to balloon temperature −30°C, s	27 (25, 30)	31 (28, 37)	<.001
Time to balloon temperature −40°C, s	32 (30, 38)	54 (45, 75)	<.001
Balloon nadir temperature (°C)	−55 (‐60, ‐51)	−47 (‐52, ‐43)	<.001
Thawing time to 0°C, s	16 (13, 20)	9 (7, 11)	<.001
PVP visible during CBA	288 (96.3%)	234 (88.6%)	<.001
TTI recorded during CBA	208 (69.6%)	167 (63.3%)	.117
TTI, s	45 (33, 69)	43 (30, 66)	.441
Balloon temperature at TTI (°C)	−46 (‐51, ‐40)	−37 (‐41, −30)	<.001
Total number of PVs	216	206	
TTI measured per PV	201 (93.1%)	164 (79.6%)	<.001

*Note*: Values are presented as median (25th, 75th percentile) or as count (%).

Abbreviations: AF, atrial fibrillation; AFA‐Pro, Arctic Front Advance Pro; CBA, cryoballoon application; PV, pulmonary vein; PVP, pulmonary vein potential; TTI, time to isolation.

^a^ Only CBA>100s was incorporated in the data.

### 3.4 Procedural and biophysical parameters associated with acute PVI

A comparison of procedural and biophysical parameters of CBAs resulting in acute PVI or no acute PVI per system is provided in Table 4. With POLARx, CBAs resulting in acute PVI were associated with higher grade of PV occlusion, lower balloon nadir temperatures, and longer thawing times till 0°C in comparison with CBAs resulting in no acute PVI. Cooling rates till −30°C or −40°C were not predictive of acute PVI when using POLARx. With AFA‐Pro, CBAs resulting in acute PVI were associated with faster cooling rates till −30°C or −40°C, lower balloon nadir temperatures, and longer thawing times till 0°C in comparison with CBAs resulting in no acute PVI.

**Table 4:** Procedural and biophysical parameters associated with acute PVI per CBA

	POLARx			AFA‐Pro		
Variables	CBA with PVI	CBA without PVI	*p*‐value	CBA with PVI	CBA without PVI	*p*‐value
Total number of CBA^a^	232	67		214	50	.349
Grade of PV occlusion						
Grade 4	208 (89.7%)	36 (53.7%)	<.001	174 (81.3%)	30 (60.0%)	<.001
Grade 3	22 (9.5%)	30 (44.8%)	<.001	39 (18.2%)	18 (36.0%)	.006
Grade 2	2 (0.9%)	1 (1.5%)	.53	1 (0.5%)	2 (4.0%)	.093
Time to balloon temperature −30°C, s	27 (25, 30)	27 (25, 30)	.807	30 (28, 35)	37 (31, 40)	<.001
Time to balloon temperature −40°C, s	32 (30, 38)	33 (30, 38)	.430	53 (43, 72)	66 (52, 91)	.015
Balloon nadir temperature_CB_ (°C)	−57 (−61, −53)	−50 (−54, −47)	<.001	−48 (−53, −45)	−43 (−47, −39)	<.001
Thawing time to 0°C, s	17 (14, 22)	13 (10, 14)	<.001	9 (8, 12)	6 (5, 8)	<.001

*Note*: Values are presented as median (25th, 75th percentile) or as count (%).

Abbreviations: AFA‐Pro, Arctic Front Advance Pro; CBA, cryoballoon application; PVI, pulmonary vein isolation.

^a^ Only CBA>100 s was incorporated in the data.

### 3.5 Complications

Overall, the incidence of complications was low in both groups. One groin hematoma occurred in the POLARx group which was treated conservatively. Two phrenic nerve palsies were recognized in each group, which did not recover at hospital discharge (Table 2). One patient in the POLARx group experienced a moderate left sided hemiparesis due to a transient ischemic attack after the procedure. CT and MRI imaging showed no demarcation of infarct areas. The patient fully recovered within 24 h without further treatment. There were no cases of cardiac tamponade, stroke, atrioesophageal fistula, and death during short‐term follow‐up.

## 4 DISCUSSION

Cryoballoon ablation has established itself as an alternative technique to radiofrequency ablation for the treatment of patients with symptomatic AF.^14^ Several randomized trials have shown noninferiority with respect to efficacy and safety of the first‐ and second‐generation cryoballoon systems in comparison with radiofrequency ablation.^2^, ^3^, ^4^, ^5^ The fourth‐generation cryoballoon (AFA‐Pro, Medtronic) has a 40% shorter distal tip (8 mm) which improves real‐time measurement of PVI. This has resulted in fewer CBAs, shorter balloon in body times and shorter procedure times in comparison to the second‐generation cryoballoon.^15^

Recently, a novel cryoballoon system, POLARx (Boston Scientific), became commercially available. Similar to AFA‐Pro, it consists of a double‐layer balloon of 28 mm and has eight refrigerant injection ports resulting in cooling of the entire distal half of its surface. Furthermore, the location of the thermocouple of the POLARx cryoballoon is similar to AFA‐Pro (21.5 mm between thermocouple and injection coil),^16^, ^17^ thus, measurements of inner balloon temperatures should be similar. The main difference between the two cryoballoon technologies is the constant pressure inside the POLARx balloon. The pressure of the POLARx is comparable with AFA‐Pro in an inflated, nonfrozen state. However, with the POLARx balloon, the pressure is kept constant even during the freeze.

Although the basic principles between both cryoablation systems are similar, we did observe a learning curve effect in our study. This can be explained by the different new features of the POLARx platform which required some time to become familiar with it. Besides changes in hardware (i.e., detailed console interface, foot pedal option, different catheter handle design, and more flexible sheath), the approach to achieve pulmonary vein occlusion is different than with AFA‐Pro. The constant balloon pressure results in a more compliant cryoballoon. To achieve optimal PV occlusion the cryoballoon should be co‐axially aligned with the PV and minimal forward push should be used. Excessive push may result in a too distal placement of the cryoballoon which will not be compensated by a pop‐out phenomenon. This is especially true for ablations of the right sided PVs to avoid phrenic nerve palsy. An advantage of the constant balloon pressure and compliant balloon is that deformations caused by pushing the balloon to obtain a good occlusion grade will be kept in size and shape during the freeze with POLARx. In addition, a more compliant balloon could theoretically provide a better balloon‐tissue contact.

Balloon‐tissue contact is important to achieve an optimal effect of cryoballoon ablation. The magnitude of PV occlusion as visualized by PV angiography is a practical marker of optimal balloon‐tissue contact and is a predictor of a durable PVI.^10^ In clinical practice, the aim is to achieve a Grade 4 PV occlusion before starting cryoablation. In our study, there was no difference in the degree of PV occlusion achieved during CBA between POLARx and AFA‐Pro. For both systems, CBAs resulting in acute PVI were associated with a higher proportion of Grade 4 PV occlusion in comparison with CBAs without acute PVI.

Several studies have shown that TTI is the most powerful predictor of durable PV isolation.^10^, ^18^, ^19^, ^20^, ^21^ In clinical practice, a TTI less than or equal to 60 s is the target for CBA. In our study, there was no difference in the median TTI between both systems and the median TTI was less than or equal to 60 s. This suggests that the speed of cryoenergy transfer to the atrial tissue is similar between both systems. Interestingly, TTI could be recorded in a higher percentage of PVs with POLARx than with AFA‐Pro (93.1% vs. 79.6%). This difference may be explained by the shorter distal tip of POLARx (5 mm) in comparison to AFA‐Pro (8 mm), which brings the circular mapping catheter closer to the PV ostium. Furthermore, there is an additional insulation of the core wire in POLARMAP (circular mapping catheter) which allows an increase in recording gain without jeopardizing the quality of the signal (higher signal‐to‐noise ratio).

Several biophysical parameters have been evaluated as possible predictors of durable PV isolation, such as balloon cooling rates, balloon nadir temperature, and balloon thawing times.^10^, ^11^, ^12^ Previous studies have shown that the most reliable biophysical marker of durable PVI is the balloon thawing time with the first‐ and second‐generation cryoballoon (Arctic Front and Arctic Front Advance, Medtronic).^10^, ^12^ Longer thawing times may not only represent colder CBA but also more effective CBA. A longer thawing time is believed to promote additional cellular injury.^10^, ^22^ The present study showed that the POLARx system has a longer thawing time till 0°C than AFA‐Pro. Whether this translates into a higher prevalence of durable PV isolation with POLARx is unknown and requires further investigation.

In our study, the POLARx system achieves faster balloon cooling rates and lower balloon nadir temperatures than AFA‐Pro. However, cooling rates till −30°C or −40°C was not associated with acute PVI with POLARx in contrast to AFA‐Pro. TTI was comparable between systems, despite lower balloon temperatures at TTI (difference of approximately 10°C) with the POLARx system. Balloon nadir temperatures was associated with acute PVI with both systems. Previous studies have shown that the balloon nadir temperature is a weak indicator for durable PVI and cooling rates are not predictive for durable PVI with the second‐generation cryoballoon (Arctic Front Advance, Medtronic).^10^, ^12^ Balloon temperatures provide an imprecise reflection of the target atrial tissue temperatures. This is not surprising as it depends on many factors including balloon positioning within the PV ostium, balloon‐to‐PV size ratio, and ipsilateral PV blood flow. Furthermore, although the location of the thermocouple is similar between both POLARx and AFA‐Pro, we cannot exclude the possibility that the more compliant balloon of POLARx may bring the thermocouple closer to the cooling area than AFA‐Pro resulting in lower balloon temperatures.

### 4.1 Study limitations

First, the data acquired from the POLARx system was based on our initial experience with this novel cryoballoon system. Although the general workflow of the procedure is similar to the AFA‐Pro system, there are small differences with regard to the approach to achieve optimal PV occlusion. This is reflected by the longer procedure and balloon in body times with the POLARx system which improved during the second phase of the study. Despite the effect of the learning curve, the total number of CBAs was similar between systems. Second, this was a nonrandomized observational study with its inherent limitations. To prevent selection bias, we used consecutive patients who underwent cryoballoon ablation. There were no significant differences in baseline variables with regard to age, sex, type of AF, and left atrial dimensions. Third, we did not systematically measure esophageal temperatures during cryoablation. Therefore, we cannot comment on the effect of the lower balloon temperatures of the POLARx system on the luminal esophageal temperature. Fourth, subclinical complications such as esophageal ulceration or PV stenosis could not be excluded, since routine diagnostic studies were not performed to investigate such complications. However, during the 1‐month follow‐up, there was no evidence of symptoms related to esophageal irritation. Lastly, we don't have information on the durability of PVI, therefore we cannot comment which biophysical parameter predicts a durable PVI with the POLARx cryoablation system.

## 5 CONCLUSION

The novel POLARx cryoballoon is comparable with AFA‐Pro with regard to efficacy and safety. Accordingly, a short learning curve is required to get used to the slightly different handling due to the compliant nature of the balloon. POLARx was associated with faster cooling rates and lower balloon temperatures but TTI was similar in both groups.

## CONFLICT OF INTERESTS

SCY is a consultant for Boston Scientific and received a research grant from Medtronic. AA is a consultant for Boston Scientific, Farapulse Inc and Galaxy Medical Inc. AL is a consultant for Biosense Webster and Boston Scientific and received speakers fee from Bristol‐Myer Squibb. The other authors have no conflict of interests.

## Data Availability

The data that support the findings of this study are available from the corresponding author upon reasonable request.

## References

[1] Hindricks G , Potpara T , Dagres N , et al. 2020 ESC Guidelines for the diagnosis and management of atrial fibrillation developed in collaboration with the European Association of Cardio‐Thoracic Surgery (EACTS). Eur Heart J. 2020.

[2] Kuck KH , Brugada J , Fürnkranz A , et al. Cryoballoon or radiofrequency ablation for paroxysmal atrial fibrillation. N Engl J Med. 2016;374:2235‐2245. 27042964 10.1056/NEJMoa1602014

[3] Luik A , Radzewitz A , Kieser M , et al. Cryoballoon versus open irrigated radiofrequency ablation in patients with paroxysmal atrial fibrillation: the prospective, randomized, controlled, noninferiority freeze AF study. Circulation. 2015;132:1311‐1319. 26283655 10.1161/CIRCULATIONAHA.115.016871 PMC4590523

[4] Buiatti A , von Olshausen G , Barthel P , et al. Cryoballoon vs. radiofrequency ablation for paroxysmal atrial fibrillation: an updated meta‐analysis of randomized and observational studies. Europace. 2017;19:378‐384. 27702864 10.1093/europace/euw262

[5] Andrade JG , Champagne J , Dubuc M , et al. Cryoballoon or radiofrequency ablation for atrial fibrillation assessed by continuous monitoring: a randomized clinical trial. Circulation. 2019;140:1779‐1788. 31630538 10.1161/CIRCULATIONAHA.119.042622

[6] Fortuni F , Casula M , Sanzo A , et al. Meta‐analysis comparing cryoballoon versus radiofrequency as first ablation procedure for atrial fibrillation. Am J Cardiol. 2020;125:1170‐1179. 32087997 10.1016/j.amjcard.2020.01.016

[7] Luik A , Kunzmann K , Hörmann P , et al. Cryoballoon vs. open irrigated radiofrequency ablation for paroxysmal atrial fibrillation: long‐term FreezeAF outcomes. BMC Cardiovasc Disord. 2017;17:135. 28545407 10.1186/s12872-017-0566-6 PMC5445510

[8] Providencia R , Defaye P , Lambiase PD , et al. Results from a multicentre comparison of cryoballoon vs. radiofrequency ablation for paroxysmal atrial fibrillation: is cryoablation more reproducible? Europace. 2017;19:48‐57. 27267554 10.1093/europace/euw080

[9] Anic A , Martin A , Breskovic T , et al. Biophysical indicators of acute pulmonary vein isolation with a novel cryoballoon. Circulation. 2020;142:A15503.

[10] Aryana A , Mugnai G , Singh SM , et al. Procedural and biophysical indicators of durable pulmonary vein isolation during cryoballoon ablation of atrial fibrillation. Heart Rhythm. 2016;13:424‐432. 26520204 10.1016/j.hrthm.2015.10.033

[11] Fürnkranz A , Köster I , Chun KRJ , et al. Cryoballoon temperature predicts acute pulmonary vein isolation. Heart Rhythm. 2011;8:821‐825. 21315836 10.1016/j.hrthm.2011.01.044

[12] Ghosh J , Martin A , Keech AC , et al. Balloon warming time is the strongest predictor of late pulmonary vein electrical reconnection following cryoballoon ablation for atrial fibrillation. Heart Rhythm. 2013;10:1311‐1317. 23792110 10.1016/j.hrthm.2013.06.014

[13] Neumann T , Vogt J , Schumacher B , et al. Circumferential pulmonary vein isolation with the cryoballoon technique results from a prospective 3‐center study. J Am Coll Cardiol. 2008;52:273‐278. 18634982 10.1016/j.jacc.2008.04.021

[14] Calkins H , Hindricks G , Cappato R , et al. HRS/EHRA/ECAS/APHRS/SOLAECE expert consensus statement on catheter and surgical ablation of atrial fibrillation. Heart Rhythm. 2017;2017(14):e275‐e444. 10.1016/j.hrthm.2017.05.012 PMC6019327 28506916

[15] Aryana A , Kowalski M , O′Neill PG , et al. Catheter ablation using the third‐generation cryoballoon provides an enhanced ability to assess time to pulmonary vein isolation facilitating the ablation strategy: Short‐ and long‐term results of a multicenter study. Heart Rhythm. 2016;13:2306‐2313. 27503480 10.1016/j.hrthm.2016.08.011

[16] Moltrasio M , Sicuso R , Fassini GM , et al. Acute outcome after a single cryoballoon ablation: Comparison between Arctic Front Advance and Arctic Front Advance PRO. Pacing Clin Electrophysiol. 2019;42:890‐896. 31046130 10.1111/pace.13718

[17] Straube F , Dorwarth U , Pongratz J , et al. The fourth cryoballoon generation with a shorter tip to facilitate real‐time pulmonary vein potential recording: Feasibility and safety results. J Cardiovasc Electrophysiol. 2019;30:918‐925. 30907462 10.1111/jce.13927

[18] Dorwarth U , Schmidt M , Wankerl M , Krieg J , Straube F , Hoffmann E . Pulmonary vein electrophysiology during cryoballoon ablation as a predictor for procedural success. J Interv Card Electrophysiol. 2011;32:205‐211. 21594628 10.1007/s10840-011-9585-x

[19] Chierchia GB , de Asmundis C , Namdar M , et al. Pulmonary vein isolation during cryoballoon ablation using the novel Achieve inner lumen mapping catheter: a feasibility study. Europace. 2012;14:962‐967. 22411731 10.1093/europace/eus041

[20] Chun KJ , Bordignon S , Gunawardene M , et al. Single transseptal big Cryoballoon pulmonary vein isolation using an inner lumen mapping catheter. Pacing Clin Electrophysiol. 2012;35:1304‐1311. 22882344 10.1111/j.1540-8159.2012.03475.x

[21] Kühne M , Knecht S , Altmann D , et al. Validation of a novel spiral mapping catheter for real‐time recordings from the pulmonary veins during cryoballoon ablation of atrial fibrillation. Heart Rhythm. 2013;10:241‐246. 23041571 10.1016/j.hrthm.2012.10.009

[22] Mazur P . Freezing of living cells: mechanisms and implications. Am J Physiol. 1984;247:C125‐C142. 6383068 10.1152/ajpcell.1984.247.3.C125

